# Meningeal lymphatic dysfunction drives cognitive impairment after experimental subarachnoid hemorrhage

**DOI:** 10.1016/j.neurot.2025.e00819

**Published:** 2025-12-13

**Authors:** Yichen Cai, Yanxin Shao, Hui Yuan, Lina Feng, Jing Wang, Mingfeng Yang, Cong Li, Baoliang Sun, Leilei Mao

**Affiliations:** a The Second Affiliated Hospital, College of Medical Information and Artificial Intelligence, Institute of Brain Science and Brain-inspired Research, Shandong First Medical University & Shandong Academy of Medical Sciences, Jinan, Shandong Province, 250117, China; b Department of Neurology, The Third Affiliated Hospital of Qiqihar Medical University, Qiqihar, Heilongjiang 161002, China; c Departmentof Anesthesiology, Shanghai Pulmonary Hospital, School of Medicine, Tongji University, Shanghai, 200433, China; d State Key Laboratory of Brain Function and Disorders, MOE Frontiers Center for Brain Science, and Institutes of Brain Science, Fudan University, Shanghai, 20032, China

**Keywords:** Subarachnoid hemorrhage, Meningeal lymphatic vessels, Cognitive impairment, PI3K-AKT pathway, VEGF-C

## Abstract

More than half of subarachnoid hemorrhage (SAH) survivors develop delayed cognitive dysfunction, but the underlying mechanisms remain elusive. This study investigated the role of meningeal lymphatic vessels (mLVs) in this complication by examining their structural integrity, drainage capacity, and association with cognitive deficits post-SAH. In adult male C57BL/6J mice in which SAH was induced by intracisternal injection of autologous blood, spatial learning and memory, and hippocampal CA1 neuronal activity were impaired as early as 1 month post-surgery, with a marked exacerbation of these deficits at 2 months. SAH induced mLV fragmentation and atrophy, subsequent cerebrospinal and interstitial fluid drainage impairment, metabolite accumulation, and ultimately delayed cognitive dysfunction. Notably, lymphatic vessel ablation exacerbated these pathologies. In vitro experiments confirmed that vascular endothelial growth factor C (VEGF-C) reduced oxyhemoglobin-induced lymphatic endothelial cell apoptosis. Furthermore, in vivo studies demonstrated that VEGF-C therapy inhibited amyloid-β (Aβ) deposition in the hippocampal CA1 region and ameliorated cognitive dysfunction. Additional studies revealed that VEGF-C's protective effect on mLVs may be mediated via PI3K-AKT pathway activation. Collectively, these findings indicate that disrupted mLV integrity and drainage contribute to post-SAH cognitive impairment. Activation of VEGF-C-mediated PI3K-AKT signaling may preserve mLV function and represent a potential therapeutic strategy for preventing delayed cognitive impairment after SAH.

## Introduction

Subarachnoid hemorrhage (SAH) is a type of stroke caused by the leakage of blood into the subarachnoid space following the rupture of blood vessels at the base or surface of the brain, accounting for 5 ​%–10 ​% of all stroke cases [1]. Approximately 500,000 individuals worldwide experience SAH each year. Among nontraumatic SAH cases, the primary cause is cerebral aneurysm rupture, which accounts for about 85 ​% of all cases. The prevalence of unruptured intracranial aneurysms is estimated to be approximately 1 in every 20 to 30 adults (3.2 ​%) [2]. The incidence of SAH increases with age, and the rate in females is about 1.3 times that in males [3]. Although the prognosis of SAH has improved over the past few decades, 12 ​% of patients still die before reaching the hospital, and the 90-day mortality rate among hospitalized SAH patients remains approximately 30 ​% [4,5]. Survivors frequently experience long-term functional and cognitive sequelae that reduce quality of life and hinder their ability to return to work. Because about half of SAH patients are under 55 years of age, many are in the most productive phases of their careers. Consequently, the economic burden is substantial because of the loss of productive years [5]. Therefore, it is critically important to actively explore therapeutic strategies targeting the sequelae of SAH.

Blood components that accumulate in the subarachnoid space and brain tissues, along with degradation products from tissue and cellular damage and various metabolites, act as pathogenic or persistent triggers for post-SAH brain injury. The mechanisms and efficiency of clearing or eliminating these substances have a major influence on the overall prognosis of SAH [6,7].The lymphatic system is an essential component of the body's defense mechanisms, contributing to the regulation of water and ion homeostasis, metabolic processes, and immune stability [8]. Studies have shown that both human and rodent brains possess a distinct cerebral lymphatic drainage system. This system comprises cerebrospinal fluid (CSF) drainage pathways, including sinus-associated meningeal lymphatic vessels (mLVs) and olfactory nerve and cervical lymph node pathways, the glymphatic system within the brain parenchyma, and perivascular pathways within the basement membrane [6,9]. The cerebral lymphatic system facilitates the entry of CSF into the brain parenchyma, enabling communication between CSF and interstitial fluid (ISF), and mediates the drainage of ISF from the brain parenchyma to adjacent lymph nodes. Through interactions with cerebral blood vessels, brain parenchyma, and the immune system, this system plays a crucial role in maintaining cerebral water and ion balance, eliminating metabolic waste, reabsorbing macromolecular solutes, facilitating the transport of signaling molecules between astrocytes, and regulating intracranial pressure. Additionally, the brain communicates with the immune system through the lymphatic drainage system, which regulates neuroimmune responses and facilitates immune surveillance [[9], [10], [11], [12]].

MLVs and olfactory and nasal lymphatic vessels are responsible for draining CSF and ISF produced within the brain parenchyma to the cervical lymph nodes. Notably, mLVs are distributed parallel to meningeal sinuses and are composed of conventional lymphatic endothelial cells. Their functions include maintaining water and ion balance in the brain and mediating the excretion of metabolites, immune cells, and other macromolecular substances into cervical lymphatic vessels. Therefore, mLVs are essential for maintaining brain tissue homeostasis and coordinating cerebral immune responses [9,13,14]. Delayed cognitive impairment is a common complication of SAH, with more than 50 ​% of survivors experiencing varying degrees of cognitive dysfunction, which severely impairs their ability to work and perform daily activities [15]. Several mechanisms have been proposed to explain post-SAH cognitive impairment, including direct or indirect neuronal and glial cell damage caused by blood and its metabolites through inflammatory and pro-apoptotic mechanisms [16]; ischemic-hypoxic injury to neurons resulting from cerebral vasospasm and delayed cerebral ischemia [17]; and histological damage secondary to microcirculatory dysfunction [18,19]. However, these mechanisms do not fully explain the long-term progressive cognitive decline observed in patients after SAH. Whether CSF drainage pathway disorders contribute to delayed cognitive impairment after SAH by impairing the clearance of toxic metabolites and cellular debris in the brain remains an important question for further investigation.

In the present study, we will investigate the occurrence of cognitive impairment within two months post-SAH and explore its correlation with the fragmentation and atrophy of mLVs, impaired drainage of CSF and ISF via mLVs, as well as the abnormal accumulation of metabolites and other macromolecules in the brain. Additionally, we will examine whether vascular endothelial growth factor-C (VEGF-C) can exert a protective effect on mLVs to improve meningeal lymphatic drainage and cognitive function post-SAH, and further explore the potential underlying protective mechanisms. These findings will represent a significant advancement in addressing the late progressive exacerbation of cognitive dysfunction following SAH and may offer an important therapeutic strategy for SAH survivors.

## Materials and Methods

### Animals

Adult male C57BL/6J mice (8–12 weeks old; 25–28 ​g) were used in this study. The mice were housed under standard laboratory conditions with a relative humidity of 50 ​± ​10 ​%, a 12-h light/dark cycle, and a temperature range of 22–25 ​°C, with ad libitum access to water and food. All experimental procedures were approved by the Institutional Animal Care and Use Committee of Shandong First Medical University (approval number W202103030172) and were conducted in accordance with the National Institutes of Health Guide for the Care and Use of Laboratory Animals. Randomization was performed using a computer-generated random number table to assign animals to experimental groups, ensuring balanced baseline characteristics across groups. A double-blinding protocol was strictly implemented, where researchers involved in animal modeling, data collection, and statistical analysis were unaware of group allocations.

### SAH model establishing

An SAH model was established in mice by invasive autologous blood injection into the cisterna magna [20,21]. Briefly, mice were anesthetized with isoflurane (3 ​% for induction and 1.5 ​% for maintenance). A total of 0.1 ​mL fresh autologous blood was aseptically collected from the femoral artery using a heparinized syringe. The animals were then positioned at a 30°head-down tilt to maximize exposure of the perioccipital membrane. Using a short 30-gauge needle, the collected autologous blood was slowly injected into the cisterna magna at a depth of 1–2 ​mm over 30 ​s. The needle was retained in place for 5 ​min post-injection, then gently withdrawn, followed by immediate surgical closure of the incision. Mice remained in the head-down position for an additional 10 ​min to facilitate the blood flow into the basal cisterns. In the sham group, the perioccipital membrane was exposed using the same procedure but without blood injection.

Neurological deficits were evaluated using the modified Garcia score at 24 ​h and 48 ​h after SAH [22]. Scoring was independently performed by two researchers, and animals with a score discrepancy of less than 2 points were included in the analysis. The assessment included autonomous movement ability (0–3 points), spontaneous limb activity (0–3 points), extension and strength of the forelimbs (0–3 points), climbing ability (1–3 points), and tactile response (1–3 points). Mice with a total score of 10–12, indicating moderate neurological impairment, were selected for subsequent experimentation. A total of 91 mice were used in this experiment, among which 5 died and 6 were excluded. The sample sizes in this study were determined based on the established protocols of our previous studies [18], with no formal power analysis performed prior to study initiation.

### MLVs ablation

Mice were anesthetized with isoflurane (3 ​% for induction and 1.5 ​% for maintenance). verteporfin (5 ​μL; Aladdin) was injected into the cisterna magna [23,24].After 15 ​min, a midline skin incision was made to expose the skull, and verteporfin was activated using nonthermal red light with a wavelength of 689 ​nm (Coherent Opal Photoactivator, Lumenis). Irradiation was applied at four sites: one at the superior sagittal sinus, one at the junction of all sinuses, and two at the left and right transverse sinuses. Each site was exposed to a light dose of 50 ​J/cm2 at an intensity of 600 ​mW/cm^2^ for 83 ​s. The scalp was then sutured, and mice were allowed to recover on a heating pad until full consciousness was regained. The SAH model was established on day 3 after surgery.

### Cognitive function test

The Morris water maze test (MWM) was used to evaluate long-term cognitive deficits, as described previously [18]. Behavioral evaluations were conducted one and two months after model establishment. During the learning phase, mice were gently placed into the water in any quadrant with their head facing the pool wall. The time required to locate the submerged platform was defined as the escape latency, with a maximum trial duration of 60 ​s. Each mouse underwent four daily training sessions for 5 consecutive days. The memory test was conducted 24 ​h after the final learning session. The platform was removed, and mice were positioned at the midpoint of quadrant III (opposite the target quadrant). Swimming behavior across all four quadrants was observed and recorded for 60 ​s, with specific quantification of the percentage of time spent in the target quadrant. Behavioral data were recorded and analyzed using the EthoVision XT Version 8.0 system (Noldus, Netherlands).

### *Ex-in vivo* electrophysiological recording of brain slices and microelectrode array (MEA) recording

Mice were euthanized 2 months post-SAH, and their brains were promptly removed. Coronal sections (350 ​μm thick) were prepared at the hippocampal level using a vibrating microtome. The sections were first incubated in artificial cerebrospinal fluid (aCSF, pH 7.4) at 32 ​°C for 30 ​min and then maintained at room temperature for 1 ​h. Subsequently, the sections were transferred to a 6-well plate compatible with the Maestro MEA extracellular electrophysiological recording system (Axion BioSystems) [25]. Each well of the MEA plate contained 64 low-impedance platinum microelectrodes (0.04 ​MΩ/electrode) with a diameter of 30 ​μm and an interelectrode spacing of 200 ​μm. Oxygenated aCSF was continuously perfused into the wells to preserve tissue viability during recording. Spontaneous neuronal activity was recorded from the hippocampal CA1 region using the Maestro Pro MEA system and AxIS software (Axion Integrated Studio Navigator 1.5, Axion Biosystems). Recordings were obtained for 1 ​min. The average firing rate was measured by counting the number of spikes per minute from the active microelectrodes corresponding to the hippocampal CA1 region, and the amplitude was recorded as the average voltage of each spike recorded within the same interval. Each brain section recorded spontaneous neuronal activity in the left and right hippocampal CA1 regions. Data were analyzed and plotted using the Neural Metric Tool version 2.2.3.

### CSF and ISF drainage capacity tests

The CSF drainage capacity of the sham and SAH groups at 2 months was evaluated using the Evans blue (EB) injection method [26].Mice were anesthetized and secured on a stereotaxic frame, and the skull was exposed. The periosteum on the skull surface was removed with 3 ​% hydrogen peroxide to visualize the fontanel. The stereotaxic coordinates for the lateral ventricle were precisely determined (anterior-posterior [AP]: −0.6 ​mm, mediolateral [ML]: 1.5 ​mm, dorsoventral [DV]: 1.7 ​mm), and 2 ​μL of 2 ​% EB solution was injected. After a 1-h circulation period, the brain, deep cervical lymph nodes (dCLNs), and superficial cervical lymph nodes (sCLNs) were harvested. Following weighing and homogenization, the samples were incubated with 50 ​% trichloroacetic acid (TCA) at 4 ​°C for 24 ​h. After centrifugation, absorbance at 620 ​nm was measured and compared with a standard curve.

To assess ISF drainage capacity in the 2-month sham and SAH groups, a tracer method was employed. As described above, 2 ​μL of Dextran, Texas Red™, 3000 ​MW, Lysine Fixable (Invitrogen) was stereotaxically injected into the brain parenchyma at coordinates (AP: 1.5 ​mm, ML: 1.5 ​mm, DV: 1.5 ​mm), with the needle retained in place for 10 ​min post-injection. After a 1-h circulation period, brains and cervical lymph nodes were collected following saline perfusion and subjected to immunostaining analysis.

### Immunofluorescence staining

Two months after SAH, the mice were euthanized and perfused sequentially with precooled saline and 4 ​% paraformaldehyde. The dura mater, brain, and cervical lymph nodes were harvested and post-fixed in 4 ​% paraformaldehyde overnight, followed by gradient dehydration in sucrose solutions (20 ​% and 30 ​%). Subsequently, 25-μm-thick sections of brain tissue and lymph nodes were prepared using a cryostat. For immunofluorescence staining, selected sections of dura mater were rinsed with PBS, blocked with 5 ​% donkey serum, and incubated with diluted primary antibodies at 4 ​°C overnight. After washing, sections were incubated with corresponding fluorescent secondary antibodies for 1 ​h at room temperature. Finally, the sections were mounted with DAPI Fluoromount-G and imaged using a Nikon laser confocal microscope. Quantitative analysis was performed using ImageJ software. Primary antibodies included anti-LYVE1 (1:200, abcam), anti-CD31 (1:200, abcam), anti-occludin (1:200, abcam), anti-MAP2 (1:200, abcam), anti-Lyve-1 (1:200, R&D), anti-*p*-AKT (1:200, selleck), anti-FLT4 (1:200, biolegend), anti-Aβ (1:200, biolegend), anti-PI3K (1:200, santa cruz), and anti-VEGF-C (1:200, santa cruz). All fluorescent secondary antibodies were purchased from Jackson ImmunoResearch (USA).

### VEGF-C treatment

After completion of the SAH operation, hydrogel-encapsulated VEGF-C at a concentration of 0.1 μg/μL was administered through the skull [24,27]. The anesthetized mice were shaved on the head, and the skin was incised along the midline to expose the skull. Using an electric drill, five small holes were carefully made—two near the bregma and three near the lambda—until only a thin layer of dura mater remained. One microliter of hydrogel-encapsulated VEGF-C (VEGF-C: MedChemExpress, China; hydrogel preparation by Qiyue Biotechnology, China) was applied to each point, and the incision was sutured after waiting for 5 ​min.

### In vitro model of hemoglobin injury and apoptosis assays

Human lymphatic endothelial cells (LECs) were purchased from BLUEFBIO Life Sciences (China). Cells were cultured in high-glucose DMEM supplemented with 10 ​% fetal bovine serum (FBS), 100 U/mL penicillin, and6, lin 100 ​μg/mL streptomycin, cultured at 37 ​°C in a humidified atmosphere of 5 ​% CO_2_. After 24 ​h of cell culture, OxyHemoglobin (OxyHb, Sigma-Aldrich, USA) with a concentration of 1 ​mmol/L was added to make the final concentration 0.01 ​mmol/L. The OxyHb model was completed after a further 48 ​h of culture. For the OxyHb ​+ ​VEGF-C model, after changing the medium at 24 ​h, different concentrations of VEGF-C (MedChemExpress, China) were added for pre-treatment for 30 ​min before adding OxyHb to make the final concentration 0.01 ​mmol/L. The control group underwent routine medium change, and the culture time was consistent with the other groups.

Cell viability was evaluated using the MTT colorimetric assay (Sigma-Aldrich) to quantify mitochondrial dehydrogenase activity. LECs were seeded into 96-well plates at a density of 1 ​× ​10^5^ ​cells/well. After the indicated treatments, 25 ​μL of MTT solution (5 ​mg/mL in PBS) was added to each well, and plates were incubated at 37 ​°C for 4 ​h. Subsequently, the supernatant was aspirated, and 150 ​μL of dimethyl sulfoxide (DMSO, Sigma-Aldrich) was added to solubilize formazan crystals by gently shaking for 10 ​min at room temperature. Optical density (OD) was measured at 570 ​nm using a microplate reader (ELX808, BioTek), and results were expressed as the percentage of viability relative to untreated controls. For apoptosis analysis, cells were harvested and washed twice with PBS. The pellets were resuspended in 100 ​μL of binding buffer and stained with 5 ​μL of Annexin V-FITC (BD Biosciences) for 15 ​min at room temperature in the dark. Subsequently, 5 ​μL of propidium iodide (PI; 50 ​μg/mL, BD Biosciences) was added, and samples were immediately analyzed using a BD FACSCalibur flow cytometer. Data were acquired and analyzed with CellQuest Pro software (BD Biosciences), and apoptotic cells were quantified based on Annexin V^+^/PI^+^ populations in dot-plot analyses.

### Bulk RNA sequencing analysis

Dura mater tissues were dissected 2 months post-SAH, snap-frozen in liquid nitrogen, and stored at −80 ​°C until further processing. Bulk RNA sequencing was performed by He Yuan Biologicals, Inc. (Shanghai, China) using an Illumina NovaSeq 6000 platform (Illumina, USA). Raw sequencing reads were demultiplexed using CASAVA v1.8.2 to generate quality-controlled paired-end reads for downstream analysis. Differential gene expression between experimental groups was conducted using DESeq2 v1.38.3, with differentially expressed genes (DEGs) identified based on |log_2_ fold change| ​≥ ​1 and adjusted P ​< ​0.05 (Benjamini-Hochberg correction for multiple testing). Gene Ontology (GO) term enrichment was performed using the topGO v2.50.0 package, while Kyoto Encyclopedia of Genes and Genomes (KEGG) pathway analysis was executed using the clusterProfiler v4.6.0 ​R package. Protein-protein interaction networks of DEGs were constructed using the STRING database (v11.5, https://string-db.org/) with a minimum interaction confidence score of 0.4, followed by visualization and analysis in Cytoscape v3.9.1.

### Statistical analysis

Statistical analyses were performed using IBM SPSS Statistics v26.0 and GraphPad Prism v10.0. Data with normal distribution were expressed as mean ​± ​standard deviation (Mean ​± ​SD). Normality was tested using the Shapiro-Wilk test. For normally distributed data, intergroup differences were compared by one-way or two-way analysis of variance (ANOVA), followed by Bonferroni post-hoc test for multiple comparisons. For non-normally distributed data, nonparametric tests were applied: the Kruskal-Wallis test was employed for three or more independent samples. Additionally, after normality testing, homoscedasticity of data from two independent groups was assessed via Levene's test. For normally distributed and homoscedastic data, the unpaired two-tailed *t*-test was used to compare intergroup differences; violation of either assumption warranted the nonparametric Wilcoxon rank-sum test as an alternative. Spearman correlation analyses were used to explore associations between different variables. Statistical significance was defined as *P* ​< ​0.05.

## Results

### SAH exposure impaired the spatial learning and memory ability and disturbed the neuronal activities in hippocampal CA1 in mice

**Fig. 1:**
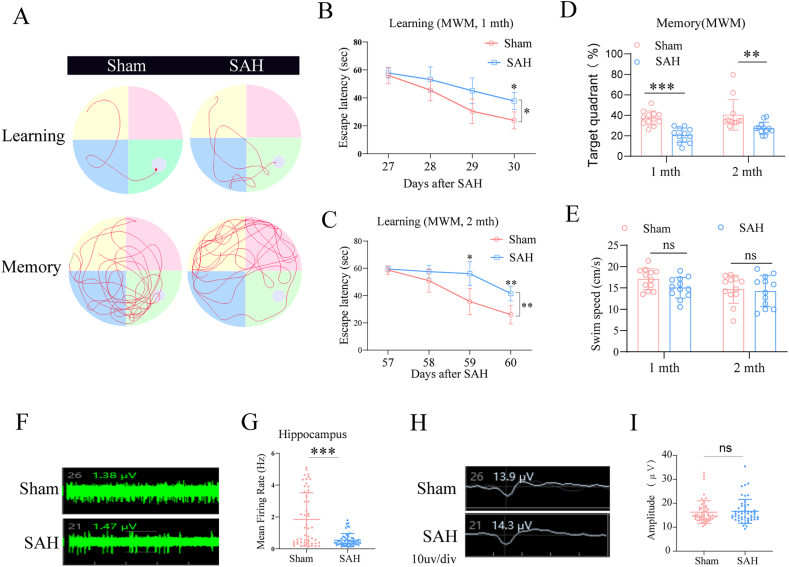
**Subarachnoid hemorrhage leads to long-term cognitive impairment and hippocampal neuronal firing reduction.** The Morris water maze (MWM) test was performed 1 and 2 months after SAH surgery. (**A)** Representative swim paths of learning and memory phase tests. (**B–C)** Time needed to reach the hidden platform in the learning phase. (**D)** The ratios of distance across the target quadrant where the platform was in the memory phase. n ​= ​12 per group. (**E)** Swimming speed after SAH. n ​= ​12 per group. (**F–I)** Frequency and amplitude of spontaneous firing of neurons in the CA1 region of the hippocampus 2 months after SAH. n ​= ​29 sham group, n ​= ​27 SAH group. (**F-G)** Representative images and quantification of the CA1 neuronal firing rate; (**H–I)** Representative images and quantification of the CA1 neuronal firing amplitude. n ​= ​12 per group, 2–3 brain sections/mice. ∗*P* ​< ​0.05, ∗∗*P* ​< ​0.01,∗∗∗*P* ​< ​0.001, ns: no significance, as indicated.

The MWM was employed to evaluate spatial learning and memory impairments in mice at 1 month and 2 months post-SAH. To assess long-term cognitive function, mice underwent a standardized MWM protocol comprising a 4-day acquisition training phase followed by a probe trial, with their movement track plots recorded (Fig. 1A) to visualize the learning and memory performance. Quantitative analysis of escape latency—the time required to locate the platform—revealed that SAH mice exhibited significantly longer latencies than sham-operated controls at both 1 month and 2 months post-SAH (Fig. 1B and C), indicating impaired spatial learning. In the probe trial, where the platform was removed, the percentage of time spent in the target quadrant was quantified. SAH mice spent a significantly smaller proportion of time in the target quadrant than sham mice at both time points (Fig. 1D). To exclude confounding variables related to motor function or anxiety, swimming speed was analyzed. No significant differences in swimming velocity were observed between SAH and Sham groups at either 1 month or 2 months post-SAH (*P* ​> ​0.05 for all; Fig. 1E).

To investigate the neurophysiological basis of SAH-induced cognitive deficits, in vitro electrophysiological recordings were performed on brain slices containing the hippocampal CA1 region at 2 months post-SAH. The results revealed that SAH mice exhibited a significant reduction in spontaneous neuronal firing rates within the CA1 pyramidal cell layer compared to sham mice (Fig. 1F and G). Additionally, no statistically significant difference was observed in firing amplitude of neuron clusters in the CA1 area between groups (*P >* 0.05*;* Fig. 1H and I). These findings suggest that exposure to SAH impairs spatial learning and memory and disrupts neuronal activity in the hippocampal CA1 region of mice.

### SAH drives disruption of meningeal lymphatic vessels integrity and subsequent cognitive impairment

**Fig. 2:**
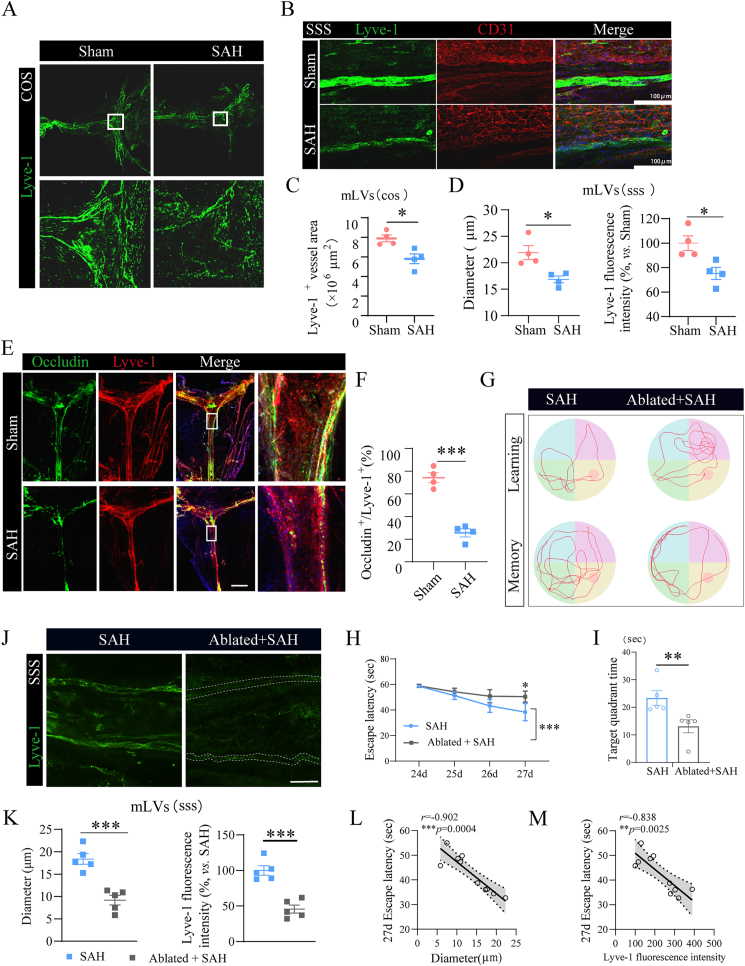
**Integrity of meningeal lymphatic vessels was impaired after SAH. (A**–**B)** Representative immunofluorescence images of meningeal sections showing Lyve-1-labeled meningeal lymphatic vessels and CD31-labeled blood vessels. (**C)** Quantitative analysis of the area fraction of Lyve-1^+^ lymphatic vessels in the confluence of sinuses (COS) region. **(D)** Quantitative analysis of the diameter of Lyve-1^+^ lymphatic vessels and Lyve-1^+^ fluorescence intensity in the superior sagittal sinus (SSS) region. n ​= ​4 per group. (**E-F)** Representative immunofluorescent images of meningeal lymphatic vessels (mLVs) co-stained for occludin and Lyve-1, accompanied by quantification of the proportion of occludin^+^ signals within Lyve-1^+^ mLVs. n ​= ​4 per group. **(G)** Representative swim paths of learning and memory phase tests. **(H)** Time needed to reach the hidden platform in the learning phase. **(I)** The ratios of distance across the target quadrant where the platform was in the memory phase. n ​= ​6 per group. **(J)** Representative immunofluorescence images of Lyve-1-labeled mLVs. **(K)** Quantitative analysis of the diameter of Lyve-1^+^ mLVs and Lyve-1 fluorescence intensity in the SSS region. n ​= ​5 per group. **(L**–**M)** Spearman correlation analysis of the 27-day escape latency and mLVs diameter (L), or Lyve-1 fluorescence intensity (M). n ​= ​10 per group. ∗P ​< ​0.05, ∗∗P ​< ​0.01, ∗∗∗P ​< ​0.001. Scale bar ​= ​100 ​μm.

MLVs are considered to be associated with intracerebral metabolism. To explore the underlying causes of long-term cognitive dysfunction following SAH, we employed immunofluorescence staining to examine the dural meningeal lymphatic vessels two months after SAH. LYVE-1, the primary receptor for hyaluronic acid (HA) in lymphatic endothelial cells, was used as a specific marker to distinguish lymphatic vessels from blood vessels (Fig. 2A), whereas CD31 was employed to label vascular endothelial cells. For detailed morphological analysis, specific anatomical regions—the superior sagittal sinus (SSS) and confluence of sinuses (COS)—were selected as regions of interest. The findings revealed that Lyve-1-positive cells on mLVs did not co-localize with CD31-expressing endothelial cells, confirming the specificity of Lyve-1 in labeling mouse brain lymphatic endothelium (Fig. 2B). Compared with the sham group, the SAH group exhibited significantly sparser Lyve-1-labeled mLVs in the COS region, along with a reduced vessel area (Fig. 2C). In the SSS region, SAH induced fragmentation and discontinuity of mLVs, accompanied by a significant reduction in lymphatic vessel diameter and Lyve-1 expression, indicative of atrophy and disruption (Fig. 2B and D). These findings indicate that the structural integrity of mLVs was compromised after SAH.

Occludin, a key tight-junction protein, plays a crucial role in blocking paracellular pathways and forming the structural foundation of these junctions. Its physiological functions primarily involve maintaining the fence function and cell barrier function. In co-labeling studies of occludin and LYVE-1, the intercellular connections of lymphatic endothelial cells within mLVs were evaluated. Quantitative analysis revealed a significant reduction in the proportion of occludin-positive lymphatic endothelial cells in the SAH group compared to the sham group (Fig. 2E and F). These results suggest that two months post-SAH, the tight connections between mLVs and lymphatic endothelial cells in the superior sagittal sinus are disrupted, leading to structural damage in the mLVs. This provides additional evidence for the compromised integrity of the meningeal lymphatic system following SAH.

Since SAH can induce disruption of mLVs integrity, we further investigated whether mLV injury drives long-term post-SAH cognitive dysfunction. Using verteporfin-mediated photodynamic ablation to induce more severe mLV damage in the SAH model, we observed more pronounced cognitive decline (Fig. 2G–I). We further verified mLV integrity in the SSS region. Compared with the SAH group, the Ablated ​+ ​SAH group showed significantly greater reductions in lymphatic diameter and Lyve-1 expression, confirming more severe mLV impairment (Fig. 2J and K). Pearson correlation analysis demonstrated a negative correlation between escape latency (27 days post-surgery) and both mLV diameter and Lyve-1^+^ expression, suggesting an association between mLV disruption extent and post-SAH cognitive outcomes (Fig. 2L-M). Collectively, our results confirm that SAH disrupts mLV integrity; this disruption, which correlates with cognitive dysfunction, may be a key contributor to post-SAH cognitive impairment.

### Meningeal lymphatic vessel injury after SAH may affect impaired substance metabolism in the brain

**Fig. 3:**
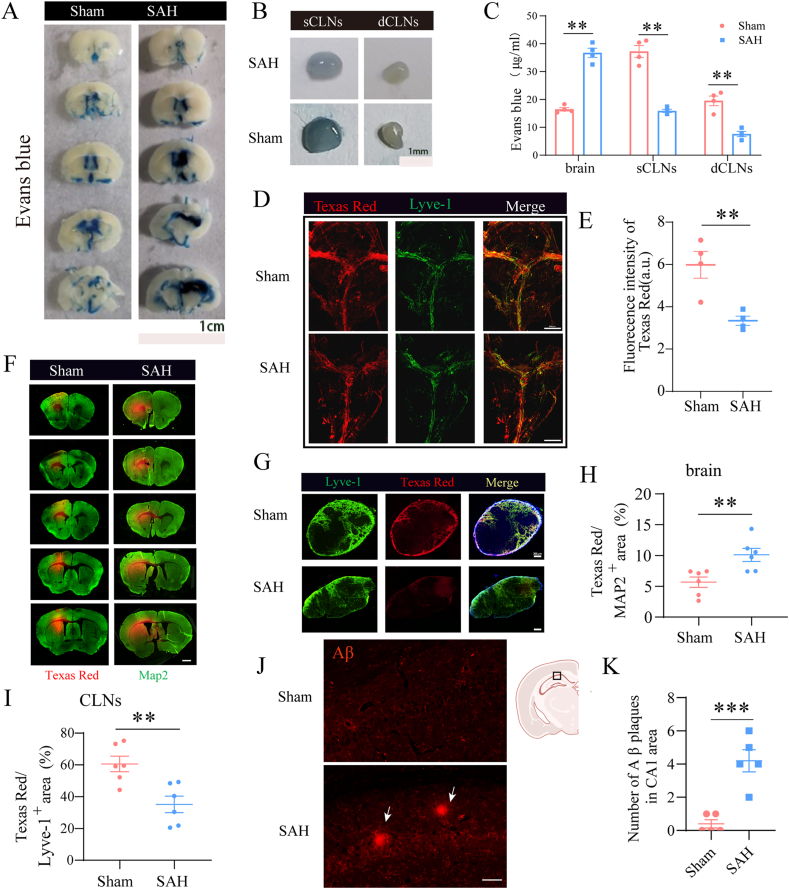
**Impaired meningeal lymphatic drainage contributes to cerebral substance metabolism dysfunction after SAH. (A**–**B)** Representative images of Evans blue (EB) distribution in brain sections, superficial cervical lymph nodes (sCLNs), and deep cervical lymph nodes (dCLNs) 1 ​h after intraventricular injection of 2 ​μl 2 ​% EB. **(C)** Quantification of EB concentrations in brain tissue, sCLNs, and dCLNs. n ​= ​4 per group. **(D**–**E)** Representative immunofluorescent images and quantification of meningeal lymphatic vessels co-labeled with Dextran, Texas Red™, 3000 ​MW, Lysine Fixable (red, tracer), and LYVE-1 (green, lymphatic marker). n ​= ​4 per group. Scale bar ​= ​2 ​mm. (**F, H)** Brain sections and dCLNs stained with MAP2 (green, neuronal marker) and Dextran, Texas Red™, 3000 ​MW, Lysine Fixable (red, tracer), with corresponding quantification of tracer distribution. n ​= ​6 per group. Scale bar ​= ​1 ​mm. **(G, I)** dCLNs stained with Lyve-1 and Texas Red, with corresponding quantification of tracer distribution. n ​= ​6 per group. Scale bar ​= ​200 ​μm. **(J)** Representative images of Amyloid β-protein (Aβ) immunostaining in the CA1 region of the hippocampus. Scale bar ​= ​100 ​μm. **(K)** Quantification of the number of Aβ plaques in the CA1 region. n ​= ​5 per group. ∗P ​< ​0.05, ∗∗P ​< ​0.01.

To assess the impact of mLVs injury on CSF drainage function following SAH, a 2-μl bolus of EB dye was stereotaxically injected into the lateral ventricle. A substantial portion of CSF can traverse the cribriform plate to enter the nasopharyngeal lymphatics, ultimately draining into the cervical lymphatic system [28]. One hour post-injection, residual EB fluorescence was quantified in the lateral ventricle, sCLNs, and dCLNs (Fig. 3A and B). The SAH group exhibited significantly increased EB accumulation in the lateral ventricle and reduced EB levels in both dCLNs and sCLNs compared to the sham group (Fig. 3C), indicating a marked decrease in mLV-mediated CSF drainage to cervical lymph nodes.

To further characterize lymphatic transport dysfunction after SAH, Dextran, Texas Red™, 3000 ​MW, and Lysine Fixable were injected into the striatal region of the brain parenchyma to trace ISF drainage. One hour following stereotaxic injection, fluorescent tracer retention was evaluated in meningeal, brain, and dCLN tissue sections. Quantitative analysis revealed significantly reduced Texas Red fluorescence intensity in mLVs of the SAH group compared to the sham group (Fig. 3D and E). Concurrently, the SAH group showed a larger area of tracer accumulation in brain parenchyma (Fig. 3F and H) and reduced tracer uptake in dCLNs (Fig. 3G and I) relative to the sham group.

Amyloid β-protein (Aβ) is recognized as a key neurotoxic substance contributing to cognitive dysfunction. An imbalance between Aβ production and clearance can lead to cognitive impairment. Next, we investigated whether the disruption of mLV integrity after SAH impairs Aβ clearance and thereby induces Aβ accumulation. Using Aβ immunofluorescence staining, we observed Aβ aggregation in the CA1 region of the hippocampus at 1 month post-SAH. This finding suggests that Aβ aggregation may be a critical factor contributing to post-SAH cognitive dysfunction (Fig. 3J and K). Collectively, these results demonstrate that SAH induces long-term impairment of CSF and ISF drainage via meningeal lymphatic pathways. This impairment further leads to Aβ metabolic dysfunction and intracerebral accumulation, which may represent a key mechanism underlying long-term cognitive dysfunction following SAH.

### VEGF-C-FLT4 interaction may be a critical determinant of mLV integrity after SAH

**Fig. 4:**
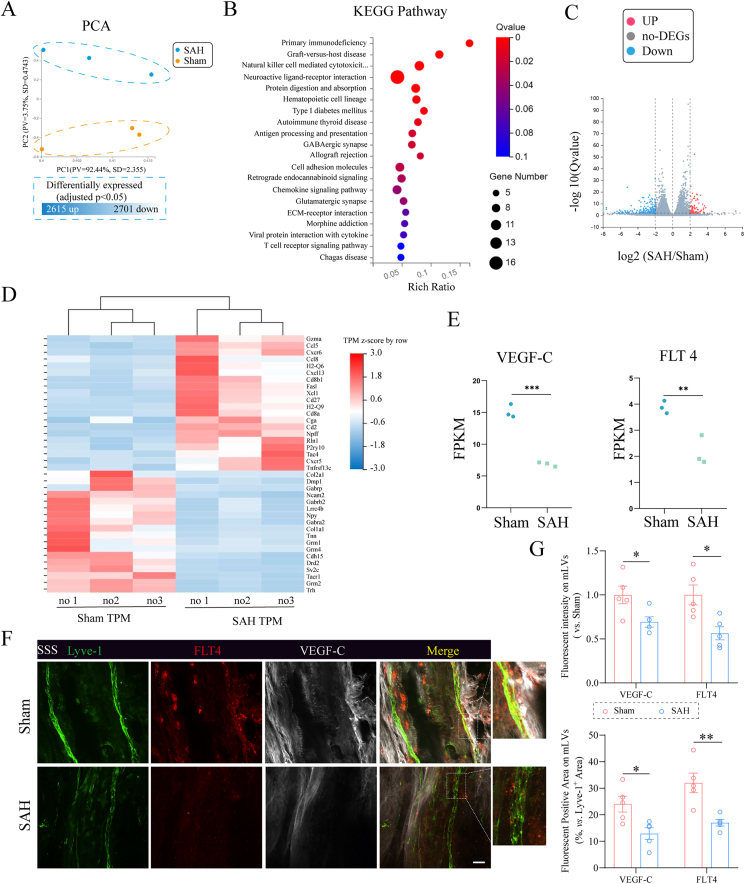
**Transcriptomic profiling of meninges following SAH. (A)** Principal component analysis (PCA) of RNA-seq data from meninges in sham and SAH groups, showing distinct clustering of transcriptomes. Differential expression analysis identified 2615 upregulated and 2701 downregulated genes in SAH meninges (adjusted Q ​< ​0.05). **(B)** KEGG pathway enrichment analysis highlighting significantly altered biological processes. **(C)** Volcano plot illustrating upregulated (red) and downregulated (blue) genes in SAH versus sham meninges. **(D)** Heatmap depicting relative expression levels of key signaling molecules and interacting genes involved in lymphatic integrity. **(E)** Quantitative expression of *VEGF-C* and *Flt4* (encoding VEGFR3) in sham and SAH meninges. n ​= ​3 mice per group. Panels (B–E) correspond to genes filtered by |log_2_FC| ​> ​2 and Q value ​< ​0.01. (**F)** Representative immunofluorescent images of mLVs co-stained for FLT4, VEGF-C and Lyve-1 in the SSS region. Scale bar ​= ​100 ​μm. **(G)** Quantification of the fluorescent intensity (upper) and positive area (lower) of VEGF-C and FLT4 on mLVs. n ​= ​5 per group. ∗P ​< ​0.05, ∗∗P ​< ​0.01,∗∗∗P ​< ​0.001.

To further investigate the effects of SAH on mLVs and elucidate the underlying molecular mechanisms, bulk RNA sequencing (RNA-seq) was performed on meningeal tissues obtained from sham-operated mice and SAH mice at two months post-surgery. Transcriptomic analysis identified 2615 upregulated and 2701 downregulated genes in SAH meninges compared to sham controls. Principal component analysis (PCA) distinctly separated the two groups, revealing significant transcriptional divergence (Fig. 4A). KEGG pathway enrichment analysis demonstrated that gene pathways including natural killer cell-mediated cytotoxicity, neuroactive ligand-receptor interaction, T cell receptor signaling pathway, chemokine signaling pathway, and cell adhesion molecules were activated, indicating abnormal functions of mLVs after SAH surgery (Fig. 4B). Filtering for genes with |log_2_FC| > 2 and a Q value ​< ​0.01 yielded a volcano plot highlighting differentially expressed genes (Fig. 4C). Heatmap analysis of selected genes showed upregulation of chemokine receptors and clusters of differentiation molecules in SAH meninges, whereas genes in cAMP and PI3K-Akt signaling pathways—such as *Col1a1, Tnn, Nyp,* and *Drd2—*were downregulated, suggesting their involvement in mLV injury (Fig. 4D).

Notably, meningeal tissues from SAH mice exhibited significant downregulation of both VEGF-C and FLT4 (VEGFR3, VEGF-C receptor) (Fig. 4E). Subsequently, we evaluated the expression of VEGF-C and FLT4 on mLVs at 1 month post-SAH using immunofluorescence staining. Compared with the Sham group, the expression levels of both VEGF-C and FLT4 on mLVs were significantly decreased following SAH (Fig. 4F–G). Given that VEGF-C/FLT4 signaling is essential for lymphatic endothelial cell proliferation and survival, these findings suggest impaired VEGF-C-FLT4 interactions may contribute to structural disruption of mLVs after SAH.

### VEGF-C treatment attenuates lymphatic endothelial cell apoptosis in an in vitro model of hemoglobin injury

Building on our previous finding that impaired VEGF-C-VEGFR3 signaling contributes to post-SAH mLV disruption, and given reports implicating VEGF-C in mLV development and maintenance [29], we investigated the cytoprotective effects of VEGF-C in an in vitro model of hemoglobin injury. Human lymphatic endothelial cells (LECs) were cultured and exposed to oxyhemoglobin to induce SAH-like injury [30], followed by treatment with recombinant VEGF-C protein.

**Fig. 5:**
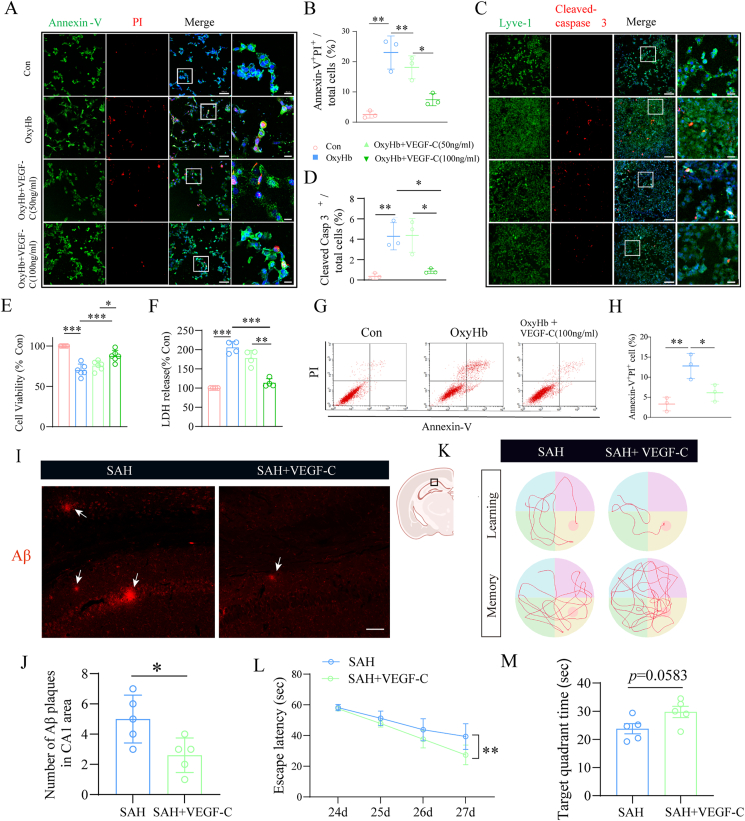
**VEGF-C inhibits the apoptosis of lymphatic endothelial cells in an in vitro model of hemoglobin injury. (A)** Representative immunofluorescence images of lymphatic endothelial cells (LECs) stained with Annexin V (viable cells, green) and PI (apoptotic cells, red). Scale bar: 100 ​μm. **(B)** Quantification of apoptotic cell percentage in LECs. n ​= ​3 per group. **(C)** Representative images of LECs co-stained with LYVE-1 (lymphatic endothelial marker, green) and cleaved-Caspase 3 (apoptotic marker, red). Scale bar ​= ​100 ​μm. **(D)** Quantification of cleaved-Caspase 3^+^ apoptotic LECs. n ​= ​3 per group. **(E)** Cell viability assessed by MTT assay (% of control). n ​= ​6 per group. **(F)** Cytotoxicity evaluated by LDH release assay (% of control). n ​= ​4 per group. **(G)** Flow cytometry scatter plots of Annexin V/PI staining: the upper right quadrant (late apoptosis) and lower right quadrant (early apoptosis) indicate apoptotic cells. **(H)** Quantification of total apoptotic cell percentage in LECs. n ​= ​3 per group. **(I**–**J)** Representative images and quantification of the number of Aβ plaques in the CA1 region of the hippocampus. n ​= ​5 per group. Scale bar ​= ​100 ​μm. **(K)** Representative swim paths of learning and memory phase tests. **(L)** Time needed to reach the hidden platform in the learning phase. **(M)** The ratios of distance across the target quadrant where the platform was in the memory phase. n ​= ​5 per group. ∗P ​< ​0.05, ∗∗P ​< ​0.01, ∗∗∗P ​< ​0.001.

Quantitative assessment of cell death and apoptosis was performed using immunofluorescent staining with PI/Annexin-V (Fig. 5A and B) and cleaved-Caspase 3/LYVE-1 (Fig. 5C and D). These analyses revealed that VEGF-C treatment at 100 ​ng/mL significantly reduced LEC death and apoptosis compared with vehicle controls, consistent with our in vivo observations. MTT assays further demonstrated that 100 ​ng/mL VEGF-C restored LEC viability, which was decreased in the SAH model, whereas 50 ​ng/mL showed no significant effect (Fig. 5E). LDH cytotoxicity assays confirmed reduced cell mortality at 100 ​ng/mL VEGF-C (Fig. 5F). Flow cytometry analysis of apoptotic LECs supported these findings, showing a marked reduction in apoptosis with 100 ​ng/mL VEGF-C treatment compared with vehicle-treated SAH models (Fig. 5G and H). Collectively, these results indicate that VEGF-C effectively inhibits lymphatic endothelial cell apoptosis in vitro, suggesting that targeting the VEGF-C-FLT4 axis may alleviate meningeal lymphatic injury and subsequent cognitive deficits following SAH.

Next, we validated the therapeutic efficacy of VEGF-C in vivo. Sustained-release VEGF-C hydrogel was administered via skull bone marrow injection following SAH, with subsequent analyses conducted at 1 month post-surgery. The results of immunofluorescence staining showed that VEGF-C treatment reduced Aβ accumulation in the hippocampal CA1 region at 1 month post-SAH (Fig. 5I and J). Cognitive function assessment using the Morris water maze test demonstrated that VEGF-C treatment significantly improved learning and memory capabilities in mice at 1 month post-SAH (Fig. 5K–M). Integrating in vitro and in vivo findings, we confirmed that VEGF-C reverses SAH-induced disruption of mLVs and exhibits substantial therapeutic potential for SAH-associated cognitive dysfunction.

### PI3K-AKT signaling pathway in the context of VEGF-C-mediated mLV protection

**Fig. 6:**
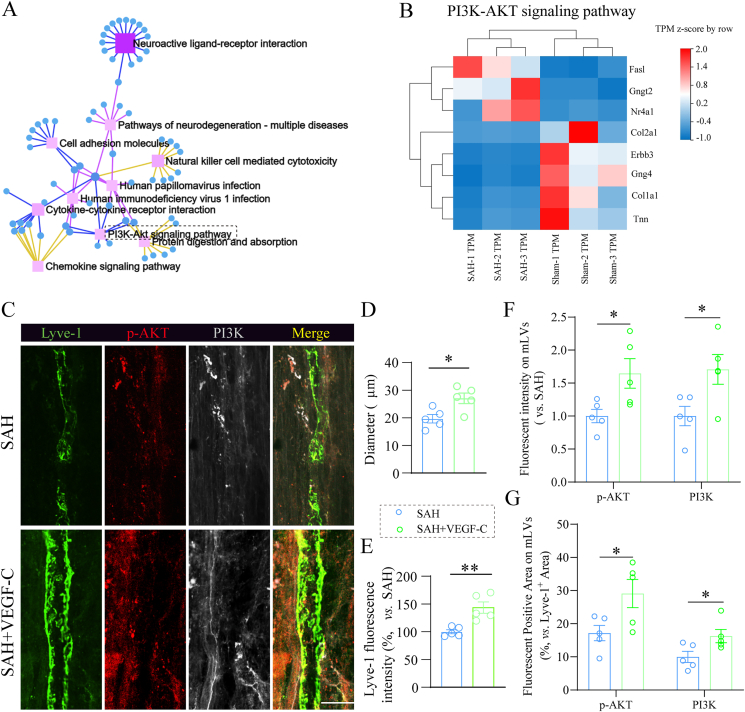
**VEGF-C-mediated protection of mLV is associated with PI3K-AKT pathway activation. (A)** KEGG pathway network analysis of differentially expressed genes (|log_2_FC| ​> ​2, Q value ​< ​0.01) illustrating their associations with canonical signaling pathways. **(B)** Heatmap depicting relative expression levels of PI3K-AKT signaling pathway-related genes in SAH versus sham meninges. n ​= ​3 mice per group. **(C)** Representative immunofluorescent images of mLVs co-stained for *p*-AKT, PI3K and Lyve-1 in the SSS region. Scale bar ​= ​50 ​μm. **(D**–**E)** Quantitative analysis of the diameter of Lyve-1^+^ lymphatic vessels and Lyve-1 fluorescence intensity in the SSS region. **(F**–**G)** Quantification of the fluorescent intensity (F) and positive area (G) of *p*-AKT and PI3K on mLVs. n ​= ​5 per group. ∗P ​< ​0.05, ∗∗P ​< ​0.01.

To further elucidate the molecular mechanisms linking VEGF-C to mLV integrity, KEGG pathway network analysis was performed on differentially expressed genes (|log_2_FC| ​> ​2, Q value ​< ​0.01) identified by RNA-seq (Fig. 6A). The integrated gene network highlighted multiple interconnected pathways, with notable enrichment in the PI3K-AKT signaling axis. Heatmap analysis of PI3K-AKT pathway-associated genes revealed coordinated dysregulation in the meninges of the SAH group compared with the sham controls (Fig. 6B). Specifically, upstream positive regulators such as *Col2a1*, *Col1a1*, *Erbb3*, *Gng4*, and *Tnn* were downregulated, whereas negative regulators including *Fasl*, *Gngt2*, and *Nr4a1* were upregulated.

Given that VEGF-C treatment effectively reverses subarachnoid hemorrhage (SAH)-induced long-term cognitive dysfunction and Aβ accumulation (Fig. 5I–M), we next assessed its protective effect on mLV integrity. The results of immunofluorescence staining showed that VEGF-C significantly increased mLV diameter and the expression of the lymphatic marker Lyve-1 in the SSS region at 1 month post-SAH (Fig. 6C–E). Furthermore, VEGF-C treatment notably elevated the fluorescence intensity of PI3K and *p*-AKT on the mLV surface, as well as the co-labeled positive area with Lyve-1^+^ lymphatic vessels (Fig. 6F–G). VEGF-C is known to activate the PI3K pathway and its downstream Akt signaling in LECs [31]. Consistent with this, our findings suggest that VEGF-C likely improves cognitive function outcomes effectively by upregulating the PI3K/AKT pathway. This pathway activation may further inhibit SAH-induced apoptosis of mLV endothelial cells and preserve mLV integrity.

## Discussion

Subarachnoid hemorrhage is a devastating cerebrovascular emergency associated with mortality and morbidity. More than 50 ​% of SAH survivors experience cognitive dysfunction, which constitutes a major long-term complication imposing significant physical, psychological, and socioeconomic burdens and compromising functional independence [5]. Despite its clinical relevance, the neurobiological mechanisms underlying post-SAH cognitive decline remain poorly understood, and no effective disease-modifying therapies are currently available. In this study, we identified structural damage to mLVs, including fragmentation and atrophy, following SAH, which was accompanied by impaired drainage of CSF and ISF. These changes may result in the accumulation of neurotoxic metabolites (eg., Aβ), thereby contributing to the development of late-stage cognitive dysfunction. In vitro and in vivo experiments further demonstrated that treatment with an optimal concentration of VEGF-C significantly reduced apoptosis in lymphatic endothelial cells in mLVs. It also effectively inhibits Aβ accumulation and ameliorates cognitive dysfunction. The results also revealed that VEGF-C exerts protective effects on mLVs potentially through activation of the PI3K-AKT signaling pathway. Collectively, these findings suggest a potential therapeutic strategy targeting the VEGF-C/PI3K-AKT axis for post-SAH cognitive dysfunction.

For decades, the brain was believed to lack a conventional lymphatic drainage system in vertebrates, including humans and rodents. However, seminal studies over the past decade have identified a specialized meningeal lymphatic network that facilitates CSF-ISF exchange. These vessels serve as critical conduits for clearing neurotoxic macromolecules (e.g., β-amyloid, hemoglobin metabolites) from the central nervous system while maintaining ion homeostasis and supporting immune surveillance [6,32]. The identification of mLVs has provided fundamental mechanistic insights into the pathophysiology of several neurodegenerative and neuroinflammatory diseases, including Alzheimer's disease, ischemic stroke, and multiple sclerosis [33,34]. In the context of SAH, blood accumulation in the subarachnoid space triggers a cascade of pathological events. Disruption of the blood-brain barrier allows entry of erythrocyte debris, metabolites, and cellular waste into the CSF [7]. We hypothesized that SAH-induced damage to mLVs and obstruction of lymphatic drainage pathways impair clearance of these toxic components, leading to their pathological accumulation in the CNS. Such dysfunction may contribute to late-stage cognitive decline after SAH, although direct mechanistic evidence remains limited. In the present study, we observed significant learning and memory deficits at 1–2 months post-SAH, coinciding with visible structural damage to mLVs (e.g., fragmentation, atrophy) and impaired lymphatic drainage to cervical lymph nodes. These alterations were associated with reduced clearance of CSF, ISF, and neurotoxic metabolites, establishing a plausible link between mLV dysfunction and progressive cognitive deterioration in the chronic phase of SAH.

VEGF-C has emerged as a critical regulator of lymphangiogenesis, particularly in meningeal lymphatic vessels that maintain central nervous system homeostasis [35]. Its protective effects on mLVs are multifaceted, encompassing dural lymphatic vessel growth, enhanced lymphatic drainage, and modulation of immune responses. In ischemic stroke models, VEGF-C-mediated dural lymphangiogenesis improves post-stroke outcomes by reducing inflammation and enhancing fluid clearance [36,37]. Age-related mLVs dysfunction correlates with reduced VEGF-C expression, and supplementation ameliorates lymphatic defects in aged mice [38]. Additionally, VEGF-C regulates neuroinflammation after SAH by facilitating meningeal lymphatic drainage of Th17 ​cells [39].In this study, we established a critical link between post-SAH cognitive dysfunction and mLV injury. RNA-seq analysis of mouse meninges at 2 months post-SAH revealed significant downregulation of both VEGF-C and Flt4 (encoding VEGFR3, the receptor for VEGF-C), suggesting that impaired VEGF-C/VEGFR3 signaling contributes to mLV damage. This hypothesis was confirmed in vitro, where recombinant VEGF-C dose-dependently attenuated apoptosis in human lymphatic endothelial cells.

The PI3K-AKT pathway is a critical intracellular signaling pathway that regulates various cellular processes, including metabolism, proliferation, cell survival, growth, and angiogenesis [40]. VEGF-C has been shown to activate this pathway, thereby exerting protective effects on lymphatic endothelial cells and promoting lymphangiogenesis [41,42]. This mechanism is particularly significant in the context of meningeal lymphatic vessels, which play a crucial role in the clearance of metabolic waste and the regulation of immune responses in the CNS [38]. Enhanced lymphatic function via VEGF-C-mediated PI3K-AKT activation has been implicated in ameliorating conditions associated with impaired lymphatic drainage, such as age-related neurodegenerative diseases [38]. In the present study, RNA sequencing analysis revealed significant inhibition of the PI3K-AKT pathway in the meninges at 2 months post-SAH. Compared with the sham control group, SAH mice exhibited coordinated dysregulation of PI3K-AKT pathway-associated genes: upstream positive regulators (e.g., *Col2a1, Col1a1, Erbb3, Gng4, Tnn*) were downregulated [[43], [44], [45]], while negative regulators (e.g., *Fasl, Gngt2, Nr4a1*) were upregulated [46,47]. These findings collectively suggest that VEGF-C may exert protective effects on mLVs through PI3K-AKT pathway activation.

Although the findings of this work advance our understanding of post-SAH cognitive impairment, several limitations should be addressed. First, our data demonstrate that VEGF-C at specific concentrations exerts protective effects on mLVs, likely through activation of the PI3K-AKT pathway. However, the precise molecular mechanisms underlying this protection remain incompletely understood. The current findings do not definitively demonstrate that the protective effect of VEGF-C on mLVs is mediated through the PI3K-AKT pathway. Therefore, in subsequent experiments, we will conduct in-depth investigations into this regulatory mechanism. Secondly, future studies should aim to validate the therapeutic efficacy of VEGF-C in mitigating mLV injury and post-SAH cognitive dysfunction in vivo, to assess whether its effectiveness is influenced by in vivo confounding factors, and to clarify cross-species applicability of rodent-derived lymphatic drainage regulators to human physiology. Finally, further investigations are warranted to identify the specific factors mediating VEGF-C's protective effects on mLVs.

SAH leads to significant cognitive dysfunction associated with structural and functional disruption of mLVs, including impaired drainage of CSF and ISF, which compromises clearance of neurotoxic metabolites, cellular debris, and macromolecules from the central nervous system. RNA-seq analysis revealed that SAH induces downregulation of Flt4, and administration of a specific concentration of VEGF-C mitigates mLVs injury post-SAH. Integrated analysis suggests that VEGF-C may protect mLVs by activating the PI3K-AKT signaling pathway. Collectively, these findings suggest that preservation of mLV integrity represents a promising therapeutic strategy for mitigating late-stage cognitive impairment after SAH.

## Author contributions

Yichen Cai and Yanxin Shao contributed equally to this work. Leilei Mao, Cong Li and Baoliang Sun designed the study. Yichen Cai, Yanxin Shao, Hui Yuan, Lina Feng, Jing Wang and Mingfeng Yang performed experiments. Yichen Cai and Yanxin Shao analyzed the data and wrote the manuscript. Leilei Mao critically edited the manuscript. All authors read and approved the final manuscript.

## Data availability

No datasets were generated or analyzed during the current study.

## Ethics approval and consent to participate

All animal experiments in this study were approved by the Animal Care and Use Committee of Shandong First Medical University (approval number W202103030172) and were performed according to ARRIVE guidelines.

## Consent for publication

Not applicable.

## Funding

This work was supported by the 10.13039/501100007129Natural Science Foundation of Shandong Province (ZR2022MH246 to LL.M., ZR2024MH100 to H.Y.), the 10.13039/100014717National Natural Science Foundation of China (82471342 to BL. S.),Y10.13039/100016697outh Innovation Technology Project of Higher School in Shandong Province (2021KJ053 to LL.M).

## Declaration of competing interest

The authors declare that they have no known competing financial interests or personal relationships that could have appeared to influence the work reported in this paper.

## Abbreviations

SAH: subarachnoid hemorrhage
mLVs: meningeal lymphatic vessels
LECs: lymphatic endothelial cells
OxyHb: oxygenated hemoglobin
CSF: cerebrospinal fluid
ISF: interstitial fluid
VEGF-C: vascular endothelial growth factor C
EB: Evans blue
dCLNs: deep cervical lymph nodes
sCLNs: superficial cervical lymph nodes
